# Genomic and epigenomic basis of breast invasive lobular carcinomas lacking *CDH1* genetic alterations

**DOI:** 10.1038/s41698-024-00508-x

**Published:** 2024-02-12

**Authors:** Higinio Dopeso, Andrea M. Gazzo, Fatemeh Derakhshan, David N. Brown, Pier Selenica, Sahar Jalali, Arnaud Da Cruz Paula, Antonio Marra, Edaise M. da Silva, Thais Basili, Laxmi Gusain, Lorraine Colon-Cartagena, Shirin Issa Bhaloo, Hunter Green, Chad Vanderbilt, Steffi Oesterreich, Anne Grabenstetter, M. Gabriela Kuba, Dara Ross, Dilip Giri, Hannah Y. Wen, Hong Zhang, Edi Brogi, Britta Weigelt, Fresia Pareja, Jorge S. Reis-Filho

**Affiliations:** 1 Department of Pathology and Laboratory Medicine, Memorial Sloan Kettering Cancer Center, New York, NY USA; 2 Department of Pathology and Cell Biology, Columbia University, New York, NY USA; 3 Department of Surgery, Memorial Sloan Kettering Cancer Center, New York, NY USA; 4 Department of Pharmacology & Chemical Biology, UPMC Hillman Cancer Center, University of Pittsburgh School of Medicine, Pittsburgh, PA USA

**Keywords:** Translational research, Breast cancer

## Abstract

*CDH1* (E-cadherin) bi-allelic inactivation is the hallmark alteration of breast invasive lobular carcinoma (ILC), resulting in its discohesive phenotype. A subset of ILCs, however, lack *CDH1* genetic/epigenetic inactivation, and their genetic underpinning is unknown. Through clinical targeted sequencing data reanalysis of 364 primary ILCs, we identified 25 ILCs lacking *CDH1* bi-allelic genetic alterations. *CDH1* promoter methylation was frequent (63%) in these cases. Targeted sequencing reanalysis revealed 3 ILCs harboring *AXIN2* deleterious fusions (*n* = 2) or loss-of-function mutation (*n* = 1). Whole-genome sequencing of 3 cases lacking bi-allelic *CDH1* genetic/epigenetic inactivation confirmed the *AXIN2* mutation and no other cell-cell adhesion genetic alterations but revealed a new *CTNND1* (p120) deleterious fusion. *AXIN2* knock-out in MCF7 cells resulted in lobular-like features, including increased cellular migration and resistance to anoikis. Taken together, ILCs lacking *CDH1* genetic/epigenetic alterations are driven by inactivating alterations in other cell adhesion genes (*CTNND1* or *AXIN2*), endorsing a convergent phenotype in ILC.

## Introduction

Invasive lobular carcinoma (ILC) is the second most prevalent histologic subtype of breast cancer, following invasive ductal carcinoma of no special type (IDC-NST), and accounts for approximately 15% of all invasive breast cancers^1,2^. The hallmark histologic feature of ILC is the discohesiveness of its neoplastic cells and the consequent growth pattern in the form of single cells and single cell files, which most commonly stem from *CDH1* biallelic inactivation^3,4^. *CDH1* maps to 16q22.1 and encodes for E-cadherin, a protein that plays pivotal roles in cell-cell adhesion mediating homophilic and homotypic adhesion in epithelial cells^4^. Bi-allelic *CDH1* inactivation is found in approximately 80% of ILCs^4,5^, in the form of *CDH1* bi-allelic mutations (i.e. a *CDH1* pathogenic mutation coupled with loss of heterozygosity (LOH) of the wild-type allele or a compound heterozygote), homozygous deletions or *CDH1* gene promoter methylation^6–8^. A subset of ILCs, however, lack *CDH1* bi-allelic inactivation, despite displaying the typical lobular phenotype^3,4^. Previous studies have suggested that alterations in genes encoding for proteins that interact with E-cadherin in the cell adhesion complex, such as α-catenin (*CTNNA1*) might be present in those cases^9^. The evidence, however, is scant, and the molecular underpinning of the lobular phenotype in ILCs lacking *CDH1* inactivation has yet to be determined.

We posited that ILCs lacking genetic or epigenetic *CDH1* alterations would be driven by inactivation of other genes playing key roles in cell-cell adhesion. Here, we sought to identify and functionally characterize the genetic alterations underpinning ILCs lacking genetic or epigenetic alterations affecting *CDH1*.

## Results

### Clinicopathologic and genomic characteristics of primary ILCs lacking inactivating *CDH1* genetic alterations

We conducted a retrospective query of 364 primary ILCs previously subjected to clinical tumor-normal targeted sequencing^10^ seeking to identify ILCs lacking *CDH1* inactivating genetic alterations. Of 364 ILCs, 314/364 (86.3%) harbored *CDH1* biallelic genetic alterations, 25/364 (6.9%) had monoallelic *CDH1* genetic inactivation, and 25/364 (6.9%) lacked *CDH1* genetic alterations. E-cadherin expression was assessed by immunohistochemistry (IHC) in 245/364 cases revealing that most ILCs displayed loss of E-cadherin expression (224/245; 91.4%), but also aberrant (12/245; 4.9%), decreased (8/245; 3.3%) and retained E-cadherin expression was observed (1/245; 0.4%; Supplementary Table S1). Notably, aberrant E-cadherin expression was found at a comparable rate (*P* = 0.52; Fisher’s exact test) across ILCs according to their *CDH1* status (Supplementary Table S1).

**Fig. 1:**
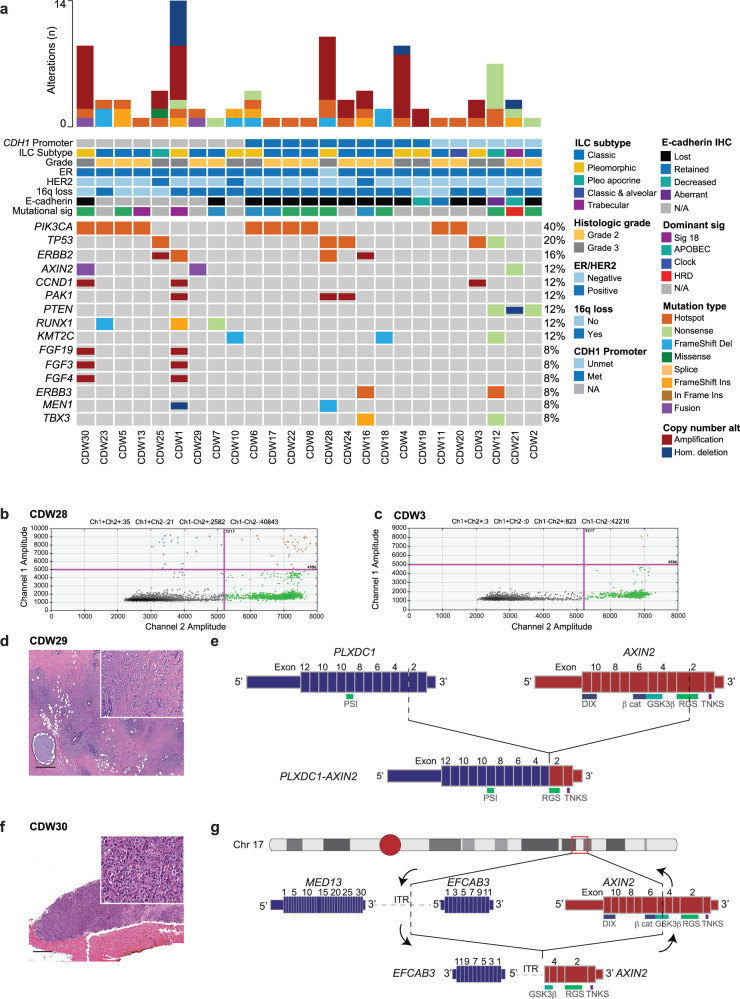
Somatic genetic alterations and *CDH1* promoter methylation in invasive lobular carcinomas lacking *CDH1* genetic alterations. **a** Nonsynonymous mutations and copy number alterations in ILCs lacking *CDH1* genetic alterations. Cases are shown in columns and genes in rows. ILC histologic subtype, histologic grade, estrogen receptor (ER) and HER2 status, chromosome 16q loss, *CDH1* promoter methylation, E-cadherin expression by immunohistochemistry, mutational signature as per SigMA, and the presence of lobular carcinoma in situ (LCIS) and/or ductal carcinoma in situ (DCIS) are shown in phenobars (*top*). **b**, **c** Representative 2D-matrix depicting methylated (Met) and unmethylated (Unmet) DNA droplets following *CDH1* promoter methylation assessment by digital droplet PCR (ddPCR). FAM probe intensity (Met) is depicted on the y-axis; HEX probe intensity (Unmet) is depicted in the x-axis. Blue (Met), orange (Met + Unmet), green (Unmet) and grey (empty). **b** Case CDW28 displays *CDH1* gene promoter methylation, and **c** case CDW3 harbors a non-methylated *CDH1* promoter. **d** Representative Hematoxylin and Eosin (H&E) micrograph of case CDW29 **e** Schematic representation of the *PLXDC1*-*AXIN2* fusion gene detected in case CDW29. Promoter, 3’UTR, 5’UTR, exons and protein domains are shown. Vertical dashed lines represent the genomic breakpoints. **f** Representative H&E micrograph of case CDW30. **g** Schematic representation a focal inversion in case CDW30. Promoter, 3’UTR, 5’UTR, exons and protein domains are shown. Vertical dashed lines represent the genomic breakpoints. β cat β catenin-binding motif, DIX DIX domain, GSK3β GSK3β-binding domain, PSI plexin repeat, RGS regulator of G protein signaling domain, TNKS tankyrase binding domain.

The 25 primary ILCs lacking *CDH1* inactivating genetic alterations included 14/25 classic ILCs (56%), 10/25 pleomorphic ILCs (40%), and one (1/25, 4%) trabecular ILC (Fig. 1a). Of these, 60% (15/25) and 40% (10/25) of cases were of histologic grades 2 and 3, respectively (Fig. 1a). Most (21/25; 84%) ILCs lacking *CDH1* inactivating genetic alterations were estrogen receptor (ER)-positive/HER2-negative, three cases (3/25; 12%) were ER-positive/HER2-positive, and one case (1/25; 4%) was ER-negative/HER2-negative. Lobular carcinoma in situ (LCIS) and ductal carcinoma in situ (DCIS) were identified in 18/25 (72%) and 4/25 (16%) of cases, respectively. Notably, in the four cases in which DCIS was present, LCIS was also identified (Fig. 1a).

The most frequently altered genes in the cohort of ILCs lacking *CDH1* inactivating genetic alterations were *PIK3CA* (10/25; 40%), affected by hotspot mutations (H1047R, *n* = 4; E542K, *n* = 3; E545K, *n* = 1; G118D, *n* = 1; M1043I, *n* = 1; N345K, *n* = 1; R88Q, *n* = 1), including two cases with 2 *PIK3CA* mutations, each, and *TP53* (5/25; 20%; Fig. 1a). Other genes found to be frequently altered in these cases were *ERBB2* (4/25; 16%), *AXIN2*, *CCND1*, *PAK1*, *PTEN*, *RUNX1* and *KMT2C* (3/25; 12%, each; Fig. 1a). Mutational signatures were inferred in those cases (15/25) with ≥ 5 single base substitutions (SBSs). Akin to previous observations in *CDH1*-mutant ILCs^8^, most cases (7/15; 53%) displayed dominant APOBEC or a clock-like/aging (4/15; 27%) mutational signatures (Fig. 1a). Lastly, we sought to compare the repertoire of somatic oncogenic/likely oncogenic genetic alterations between ILCs lacking *CDH1* inactivating genetic inactivation and ILCs harboring biallelic *CDH1* inactivating genetic alterations. Besides *CDH1*, our analyses revealed no statistically significant differences between the two groups (Supplementary Fig. 1a). In addition, we found a comparable tumor mutation burden (TMB; *P* = 0.07; Mann-Whitney *U* test) and levels of chromosomal instability as inferred by the fraction of genome altered (FGA; *P* = 0.12; Mann-Whitney *U* test) between the two groups (Supplementary Figs. 1b, c).

### *CDH1* promoter methylation in ILCs lacking *CDH1* inactivating genetic alterations

**Fig. 2:**
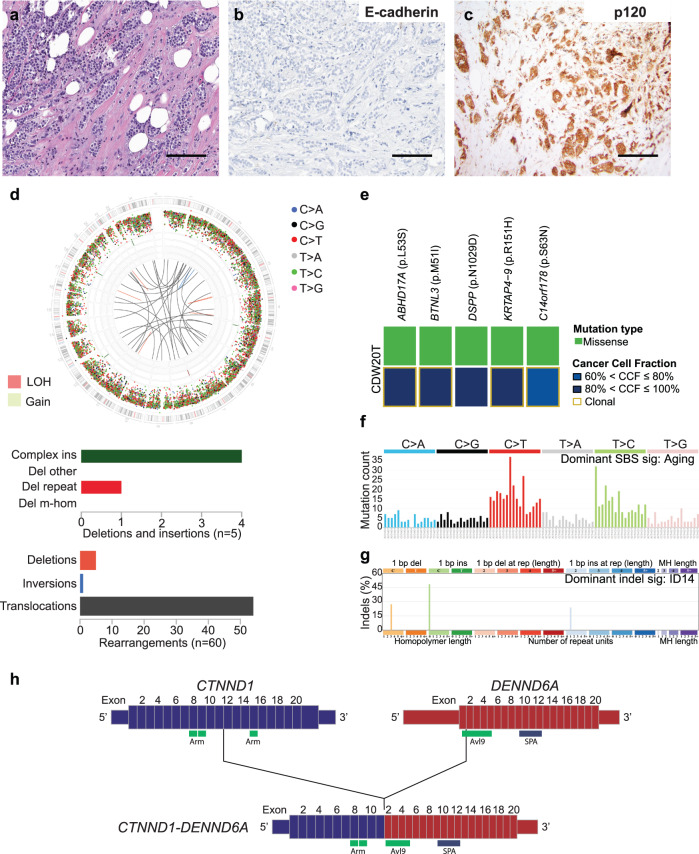
Deleterious fusion gene affecting *CTNND1* in invasive lobular carcinoma detected by whole-genome sequencing. **a–c** Representative **a** hematoxylin-and-eosin (H&E) micrographs, and micrographs depicting **b** E-cadherin expression and (**c**) p120 expression by immunohistochemistry of case CDW20. Scale bars, 100 μm. **d** Circos plot summarizing the whole-genome sequencing (WGS) findings of case CDW20 (*top*); in the circos plots, from outside to inside inter-variant distance and type of single base substitution (SBS), indels, copy number alterations and structural variants. Rearrangements (*middle*) and deletions and insertions (*bottom*) are shown. **e** Heatmap showing the non-synonymous somatic mutations and corresponding cancer cell fraction (CCF) identified in CDW20 by WGS. (**f**) SBS and (**g**) indel mutational signatures detected by Signal. **h** Schematic representation of the *CTNND1*-*DENND6A* fusion gene identified in case CDW20 by WGS. Vertical dashed lines represent the genomic breakpoints in chromosomes 11 and 3.

**Fig. 3:**
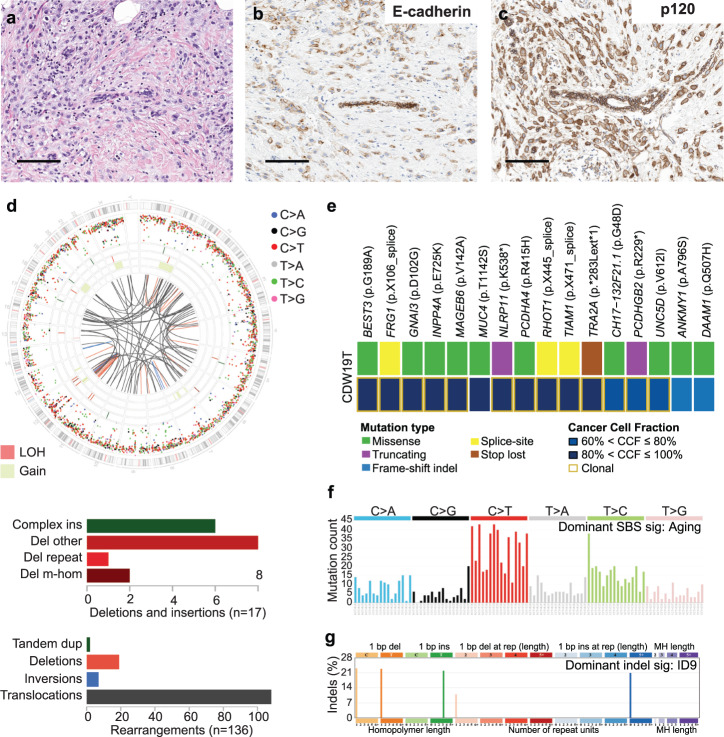
Invasive lobular carcinoma lacking *CDH1* alterations and showing *CDH1* promoter methylation without 16q loss. **a** Representative hematoxylin-and-eosin (H&E) micrographs, and micrographs depicting **b** E-cadherin expression and **c** p120 expression of case CDW19 by immunohistochemistry. Scale bars, 100 μm. **d** Circos plot summarizing the whole-genome sequencing (WGS) findings of case CDW20; in the circos plots, from outside to inside inter-variant distance and type of single base substitution (SBS), indels, copy number alterations and structural variants. Rearrangements (middle) and deletions and insertions (bottom) are shown. **e** Heatmap depicting the non-synonymous somatic mutations and corresponding cancer cell fraction (CCF) identified in case CDW19 by WGS. **f** SBS and (**g**) indel mutational signatures as detected by Signal.

**Fig. 4:**
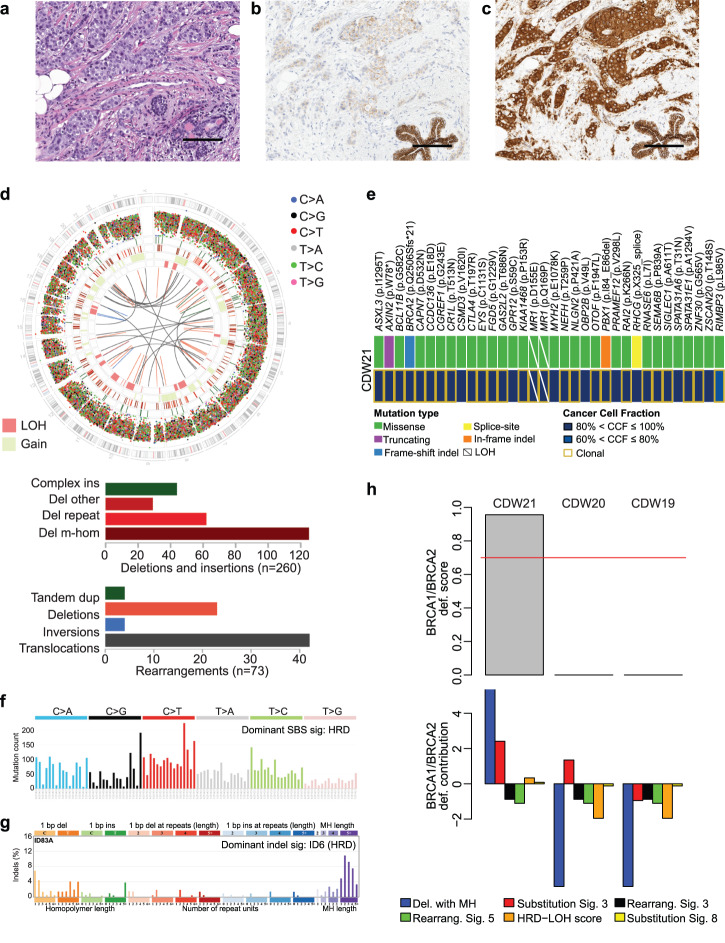
*AXIN2* inactivating mutation in invasive lobular carcinoma lacking *CDH1* genetic/ epigenetic alterations. **a** Representative hematoxylin-and-eosin (H&E) micrographs, and micrographs depicting **b** E-cadherin expression, and **c** p120 expression by immunohistochemistry of case CDW21. Scale bars, 100 μm. **d** Circos plot summarizing whole-genome sequencing (WGS) findings of case CDW21 (*top*); in the circos plots, from outside to inside inter-variant distance and type of single base substitution (SBS), indels, copy number alterations and structural variants. Rearrangements and deletions and insertions are shown (*bottom*). **e** Heatmap showing the non-synonymous somatic mutations and corresponding cancer cell fraction (CCF) identified in case CDW21 by WGS. **f** SBS mutational signature and **g** indel mutational signature in case CDW21 as identified by Signal. **h** Homologous recombination deficiency (HRD) assessment of cases CDW21, CDW19 and CDW20 using HRDetect.

To determine whether the lobular phenotype in ILCs lacking *CDH1* inactivating genetic alterations would be caused by epigenetic inactivation of this gene, we conducted the assessment of *CDH1* promoter methylation status by methylation-specific PCR (MSP) and/or digital droplet PCR (ddPCR) in all cases with available formalin-fixed paraffin-embedded (FFPE) material (*n* = 16; see Methods). These analyses revealed that 10/16 (62.5%) cases displayed *CDH1* promoter methylation, whereas 6/16 (37.5%) did not (Fig. 1a–c). Notably, all but one (9/10) of the cases showing *CDH1* promoter methylation displayed concomitant 16q loss, along with complete loss of E-cadherin expression by IHC, suggesting that both alleles of *CDH1* were inactivated in these cases (Fig. 1a). Of note, one (1/10) case (CDW19) harbored *CDH1* gene promoter methylation without associated 16q loss, and displayed decreased but not completely absent, E-cadherin expression by IHC (Fig. 1a). Three of 6 cases without *CDH1* gene promoter methylation showed complete loss of E-cadherin expression by IHC, while the other 3 cases displayed aberrant (*n* = 1), decreased (*n* = 1) or retained (*n* = 1) E-cadherin expression (Fig. 1a). Notably, 6/10 cases displaying *CDH1* promoter methylation corresponded to classic ILCs, whereas most (4/6) of the ILCs lacking *CDH1* promoter methylation corresponded to ILC variants, including two pleomorphic ILCs, one of which also displayed apocrine features, a trabecular ILC and a classic/ alveolar ILC (Fig. 1a and Figs. 2–4**)**.

No statistically significant differences in terms of histologic subtype or frequency of somatic genetic alterations between cases displaying *CDH1* gene promoter methylation (*n* = 10) and those with a non-methylated *CDH1* promoter (*n* = 6) were observed (Fig. 1a). Notably, no differences in the mutational frequency in *TP53*, whose expression has been associated to *CDH1* promoter methylation in non-lobular tumors^11,12^, were detected between the two groups (Fig. 1a). Given the low number of cases in this comparison, however, these negative results should be interpreted with caution, as type II or β errors cannot be excluded.

We sought to determine the presence of genetic alterations in genes playing pivotal roles in cell adhesion in cases lacking *CDH1* genetic or epigenetic alterations. The reanalysis of MSK-IMPACT targeted sequencing data revealed three cases (CDW21, CDW29 and CDW30) with inactivating genetic alterations affecting *AXIN2* that encodes for a multifaceted protein playing key roles in cell adhesion and differentiation^13^. CDW21 was a trabecular ILC displaying reduced E-cadherin expression which harbored a loss-of-function mutation affecting *AXIN2* (Fig. 1a). Case CDW29 was a classic ILC harboring a *PLXDC1*-*AXIN2* fusion gene resulting in the deletion of exons 3-13 of *AXIN2* with loss of the DIX domain, and the β catenin- and GSK3 β- interacting domains, as well as truncation of the regulator of G protein signaling (RGS) domain (Fig. 1d, e). Case CDW30 was a pleomorphic ILC harboring an inversion focal inversion in the long arm of chromosome 17 with breakpoints in exon 5 of *AXIN2* and an intergenic region in 17q, resulting in truncation of *AXIN2* in exon 5 with ensuing the loss of the DIX and β catenin binding domains and truncation of the GSK3 β interacting domain (Fig. 1f, g). No material was available for cases CDW29 and CDW30 and *CDH1* promoter methylation assessment was not conducted. No LOH of the wild-type allele was observed in any of the three ILCs with inactivating *AXIN2* alterations. Haploinsufficiency of *AXIN2* has been well documented, however^14–16^.

### New molecular mechanisms underpinning the lobular phenotype identified by whole-genome sequencing

We posited that ILCs lacking *CDH1* genetic or epigenetic alterations could be underpinned by the inactivation of genes involved in the cell-cell adhesion and/or by structural rearrangements not captured by targeted sequencing. To determine the molecular basis of their lobular phenotype, we subjected three cases to whole-genome sequencing (WGS), including two ILCs lacking genetic or epigenetic *CDH1* alterations (cases CDW20 and CDW21) and case CDW19, which showed *CDH1* promoter methylation in the absence of 16q loss (Fig. 1a).

Case CDW20 corresponded to a grade 2 classic ILC with focal alveolar growth, showing complete loss of E-cadherin expression and displaying cytoplasmic expression of p120 by IHC (Fig. 2a–c). WGS revealed no mutations in cell-cell adhesion genes. A clock-like/aging SBS mutational signature, along with an indel signature ID14, of cryptic origin was identified (Fig. 2d–g). Structural variant analysis revealed a translocation t(3;11) predicted to result in a deleterious fusion gene affecting *CTNND1*, which encodes for p120 (Fig. 2h), validated by reverse transcription-polymerase chain reaction (RT-PCR; see Methods) This translocation was predicted to result in the fusion of intron 11 of *CTNND1* with exon 2 of *DENND6A*, resulting in the loss of exons 12 to 20 of *CTNND1*, which encode for key armadillo repeat domains, essential for the function of p120 and stabilization of the *CDH1* complex^17^, which constitutes the likeliest basis for the lobular phenotype in this case. No LOH of the wild-type allele of *CTNND1* was observed; this gene, however, is known to be happloinsufficient^18^.

Case CDW19 corresponded to grade 3 pleomorphic ILC showing reduced, although not completely absent, expression of E-Cadherin, as well as membranous and cytoplasmatic expression of p120 (Fig. 3a–c). These findings are in line with the observation of *CDH1* gene promoter methylation without concurrent 16q loss (Fig. 1a). WGS analyses did not reveal mutations in cell adhesion genes in this case and showed aging/clock-like SBS mutational signature and an ID9 (cryptic origin) indel mutational signature (Fig. 3f, g). The most parsimonious cause for the lobular phenotype in this case is that promoter methylation affected one allele of *CDH1*, while the other allele was not affected genetically or epigenetically, resulting in partial reduction, but not complete loss, of E-cadherin expression (Fig. 1a**;** Fig. 3a–c).

Case CDW21 corresponded to a trabecular ILC of histologic grade 2 displaying markedly reduced expression of E-Cadherin, along with cytoplasmic p120 expression (Fig. 4a–c). WGS analysis validated the presence of the clonal W78* mutation affecting *AXIN2*, expected to result in a truncated protein product, identified by targeted sequencing, and showed no genetic alterations in other cell-cell adhesion genes. No LOH of the wild-type allele of *AXIN2* was observed; haploinsufficiency, however, is a well-documented phenomenon for this gene^14–16^. In addition, a *BRCA2* Q2506Sfs*21 somatic mutation associated to LOH (Fig. 4d, e) was detected, and this case displayed a dominant SBS homologous recombination (HR) DNA repair deficiency (HRD) mutational signature, a dominant ID6 (HRD) indel mutational signature, with enrichment in deletions with microhomology (Fig. 4f, g), an HRDetect score of 0.99, consistent with HRD (Fig. 4h). Taken together, case CDW21 corresponds to an HR-deficient ILC, whose lobular phenotype was likely driven by the loss-of-function of *AXIN2*.

### Loss of *AXIN2* results in the acquisition of lobular-like features in ER-positive breast cancer cell models

**Fig. 5:**
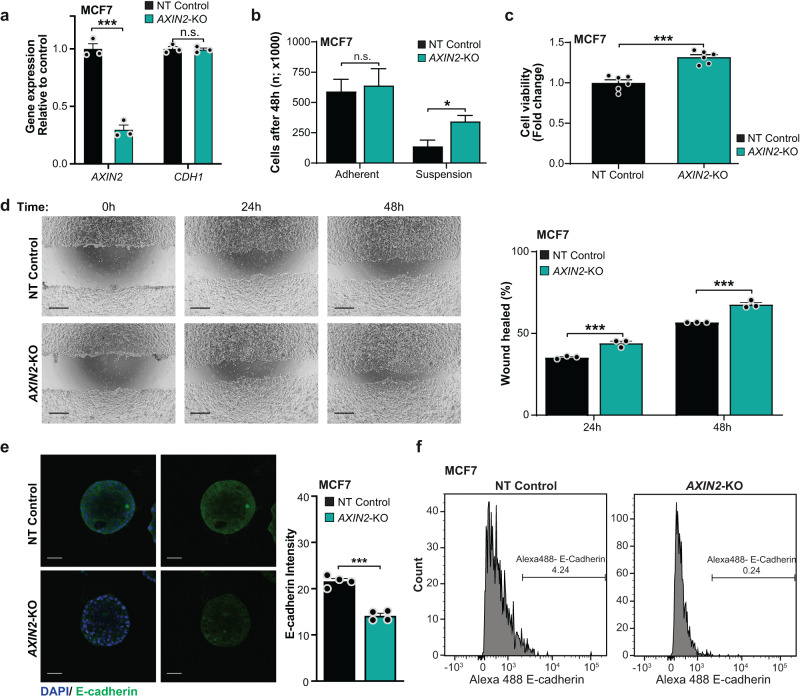
*AXIN2* loss-of-function results in the acquisition of lobular-like features in breast cancer cell models. **a** Quantitative assessment of *AXIN2* and *CDH1* mRNA expression in *AXIN2*-knock out (KO) and non-target (NT) control MCF7 cells by RT-PCR. Expression levels were normalized to *GAPDH* expression, and comparisons of mRNA expression levels were performed relative to NT control cells. **b** Number of *AXIN2*-KO and NT control MCF7 cells cultured in adherent and non-adherent (suspension) conditions for 48 hours. **c** Cell viability assay of NT control and *AXIN2*-KO MCF7 cells after culture in non-adherent conditions. **d** Wound healing assay of *AXIN2* KO and NT control MCF7 cells. Wound area was assessed at 0, 24 and 48 hours. Representative micrographs (*left*) and wound closure quantification (*right*) are shown. Scale bar, 500 μm. **e** Representative confocal micrographs of E-cadherin expression by immunofluorescence analysis of *AXIN2*-KO and NT control MCF7 3D cell models (*left*). E-cadherin (green) and 4–6-diamidino-2-phenylindole (DAPI, blue). Scale bars, 50 μm. Quantification of E-cadherin average intensity (*right*). **f** Representative flow cytometry analysis histograms of cell surface E-cadherin expression in NT control (*left*) and *AXIN2*-KO (*right*) MCF7 cells. Data are representative of at least three independent experiments. Student’s t-test; ***, *P* < 0.001; *, *P* < 0.05; n.s., nonsignificant.

Given the identification of a loss-of-function (LOF) mutation and deleterious structural variants affecting *AXIN2* in ILCs lacking *CDH1* genetic and/or epigenetic alterations and without evidence of LOF mutations in other cell adhesion genes, as well as the evidence linking *AXIN2* inactivation to cell adhesion, we sought to determine whether functional inactivation of this gene would result in the acquisition of lobular-like features in biologically relevant cell models. We employed the luminal A, ER-positive/HER2-negative breast cancer cell line MCF7, which retains E-cadherin expression^19^, and that expresses higher levels of *AXIN2* compared to other ER-positive/HER2-negative breast cancer cells, such as CAMA1 and T47D (Supplementary Fig. S2). To determine whether *AXIN2* LOF would result in the acquisition of lobular-like features, we knocked-out (KO) *AXIN2* in MCF7 cells using three single guide RNAs (sgRNAs). Out of the 3 sgRNAs tested, sgRNA2 that targeted exon 2 of this gene, was the most efficient, with 99.5% of the DNA sequenced found to be mutated, and 94.5% of the mutations being frameshifting, as assessed by CRISPR-sequencing, and validated by Sanger sequencing (Supplementary Figs. S3a-S3b). Consistent with these observations, *AXIN2* expression was reduced by approximately 70-80% in *AXIN2*-KO MCF7 cells compared to non-target (NT) control cells, as assessed by RT-PCR (Fig. 5a).

We then sought to determine whether MCF7 cells in which *AXIN2* had been knocked-out would acquire lobular-like features. Whilst no differences in cell viability were observed in adherent conditions, in low attachment (suspension) conditions, as compared to NT controls, *AXIN2*-KO cells showed increased cell growth and increased viability after being reseeded in adherent conditions following 72 hours of cell culture in low attachment conditions (Fig. 5b, c and Supplementary Fig. S4). These findings were akin to our observations upon transient silencing of *CDH1* in MCF7 cells, which resulted in increased cell viability in low attachment conditions, and increased cell viability following reseeding in adherent conditions after culture in low attachment conditions for 72 hours (Supplementary Figs. S5a-S5c). These findings are consistent with the notion that *AXIN2* LOF results in resistance to anoikis, a cardinal biologic feature of ILC^20,21^. Moreover, a wound healing assay revealed increased cellular migration in *AXIN2*-KO MCF7 cells compared to NT controls (Fig. 5d), akin to our observations upon silencing of *CDH1* (Supplementary Fig. S5d).

Although we did not observe differences in number, size, or morphology between *AXIN2*-KO and NT control MCF7 cells grown in 3D cell culture, *AXIN2*-KO MCF7 3D structures displayed decreased E-cadherin expression compared to NT controls (Fig. 5e and Supplementary Fig. S6). In line with these findings, we observed reduced E-cadherin cell surface expression in *AXIN2*-KO cells, compared to NT controls, by flow cytometry analyses (Fig. 5f), despite the comparable *CDH1* mRNA expression levels in *AXIN2*-KO and NT control cells (Fig. 5a). Notably, *CTNND1* (p120) mRNA expression was found to be comparable between *AXIN2*-KO and NT control MCF-7 cells, and between cells in which *AXIN2* had been transiently silenced with siRNAs compared to siRNA control (Supplementary Fig. S7a). Moreover, no differences in p120 protein levels were detected in *AXIN2*-KO cells compared to NT control cells (Supplementary Fig. S7b).

## Discussion

*CDH1* LOF and the ILC phenotype represent a strong genotypic-phenotypic correlation in breast cancer. Approximately 80% of ILCs harbor bi-allelic *CDH1* genetic inactivation; however, bi-allelic alterations of this gene are detected in <1% of other forms of invasive breast cancer. The basis for the lobular phenotype in the remaining cases, however, is largely unknown. In this study, we observed that a significant proportion (63%) of ILCs lacking *CDH1* genetic alterations display bi-allelic inactivation of this gene via *CDH1* gene promoter methylation associated with 16q loss. It has been previously reported that *CDH1* promoter methylation is infrequent in ILCs^22^. Here we observe, however, that among *CDH1* wild-type ILCs, *CDH1* promoter methylation is prevalent. We identified one pleomorphic ILC displaying *CDH1* promoter methylation in the absence of concurrent 16q loss; this case showed partial reduction of E-cadherin expression, along with p120 cytoplasmic and membranous expression, in contrast the ILCs displaying *CDH1* promoter methylation associated with 16q loss that displayed a complete loss of E-cadherin expression. It is possible that mono-allelic inactivation of *CDH1*, such as observed in this case, occurs in a subset of ILCs, and results in an incomplete lobular phenotype.

Mutational signatures analysis revealed that in *CDH1* wild-type ILCs, akin to *CDH1*-altered ILCs^8^, the APOBEC mutagenesis process, as defined by the APOBEC mutational signature is a common biological phenomenon. APOBEC mutagenesis has been shown to be associated with activity of the APOBEC family of cytidine deaminases^23,24^, hypermutation and drug resistance^25^. HRD analysis using WGS data and HRDetect and mutational signature analysis revealed bi-allelic *BRCA2* inactivation and HR-deficiency in an ILC lacking *CDH1* genetic/epigenetic inactivation. These findings suggest that HRD might be operative in a subset of ILCs and are in line with a reported association between *BRCA2* germline variants and ILC^26^.

Reanalysis of MSK-IMPACT data revealed the presence of deleterious fusion genes and a truncating mutation affecting *AXIN2*, which plays essential roles in various intracellular pathways as Wnt, TGFβ and Hippo pathway that regulate different biological processes, including cell adhesion, survival and differentiation^27^. WGS analysis of ILCs lacking *CDH1* genetic or epigenetic inactivation not only confirmed the *AXIN2* pathogenic mutation, but also ruled out the presence of germline or somatic genetic alterations affecting cell adhesion genes in this case. We observed that *AXIN2* depletion in biologically relevant in vitro cell models resulted in increased cellular migration, as well as increased ability to grow in non-adherent conditions, hallmark features of ILC, characterized by anchorage independent growth, anoikis resistance and a discohesive growth pattern^20,21,28–31^. Although resistance to anoikis is not exclusively linked to a lobular phenotype, it plays key roles in the induction of anchorage independence in ILC, as reported in mouse models in which *TP53* and *CDH1* inactivation resulted in primary and metastatic ILC^20,32^. These findings suggest that *AXIN2* depletion might underpin the lobular phenotype in a subset of ILCs. The WGS analysis also led to the identification of another likely alternative mechanism for the lobular phenotype, including a deleterious fusion affecting *CTNND1* (p120), a protein that directly interacts with E-cadherin. These findings support the notion that the molecular mechanisms underpinning ILC involve the disruption of the cell-cell adhesion machinery through various converging mechanisms.

Our study has important limitations, such as the limited number of cases lacking *CDH1* genetic or epigenetic alterations subjected to WGS, and the fact that epigenetic alterations in other cell adhesion genes besides *CDH1* were not interrogated. Further studies investigating in a more comprehensive manner the epigenome of ILCs lacking genomic or epigenomic *CDH1* bi-allelic inactivation are warranted. Moreover, protein expression analysis in cases with *AXIN2* alterations could not be conducted, largely due to the unavailability of material. Evaluation of AXIN2 expression in cases harboring inactivating *AXIN2* genetic alterations is warranted.

Our study indicates that the lobular phenotype in ILCs lacking *CDH1* genetic or epigenetic alterations might be driven by genetic alterations affecting alternative drivers pertaining to epithelial cell-cell adhesion. These findings support the notion that ILCs constitute a convergent phenotype, driven by disruption of the cell-cell adhesion complex, through different molecular mechanisms. The systematic analysis of a larger cohort of ILCs lacking genetic and epigenetic *CDH1* inactivation is warranted.

## Methods

### Study cases and histopathologic assessment of cases

This study was approved by the Institutional Review Board (IRB) of Memorial Sloan Kettering Cancer Center (MSK). This study is in compliance with the Declaration of Helsinki. Written informed consent was obtained according to IRB protocols. We investigated the presence of inactivating *CDH1* genetic alterations through the reanalysis of targeted sequencing data corresponding to 364 primary ILCs that had been previously subjected to clinical paired tumor/normal targeted sequencing using the FDA-authorized MSK-IMPACT assay^10^, to identify cases lacking *CDH1* bi-allelic genetic alterations. Histopathologic review of ILCs lacking *CDH1* bi-allelic genetic inactivation was conducted by six pathologists with expertise in breast pathology (FD, LCC, DG, HZ, FP and JSR-F) following the criteria put forward by the World Health Organization (WHO)^33^. Cases were graded following the Nottingham grading system^33,34^. Estrogen receptor (ER) and HER2 status were retrieved from the medical charts.

### Immunohistochemical analysis

E-cadherin expression was assessed by IHC in all cases with available FFPE material and p120 expression was assessed by IHC in a subset of cases. IHC staining for E-cadherin (clone 36, Ventana, Tucson, AZ) and/or p120 (clone 98, Ventana) was conducted with appropriate positive and negative controls included in each slide run, with antibody solutions ready to use. Expression and localization of E-cadherin and p120 was reviewed by two pathologists (FD and FP). E-cadherin expression was categorized as negative, retained, aberrant or reduced, as per Da Silva et al.^35^ and Choi et al^36^. Expression of E-cadherin was considered aberrant if reactivity was discontinuous membranous, cytoplasmic and/or dot/like/granular. E-cadherin expression was considered decreased if membranous reactivity was reduced, compared to surrounding normal epithelium.

### DNA extraction

Tumor and normal tissue blocks were retrieved from the MSK Pathology archives. Ten to 15 eight-µm-thick representative formalin fixed paraffin-embedded (FFPE) tumor and matching normal tissue sections were stained with nuclear fast red and microdissected using a sterile needle under a stereomicroscope (Olympus SZ61) to ensure a tumor content >70%, as previously described^37^. Genomic DNA was extracted using the DNeasy Blood and Tissue Kit (Qiagen) according to manufacturers’ instructions^38^.

### *CDH1* promoter methylation assessment by methylation-specific PCR

Tumor derived genomic DNA (50–250 ng) of samples with available FFPE material was subjected to bisulfite conversion using the EZ DNA Methylation-Lightning Kit (Zymo Research; Irvine, CA), following the manufacturers’ instructions^39^. Methylation-specific PCR (MSP) was conducted to assess the promoter methylation status of *CDH1*. The following primers were used for amplification of a fragment corresponding to the *CDH1* methylated sequence: forward, 5′-TTAGGTTAGAGGGTTATCGCGT-3′ and reverse, 5′-TAACTAAAAATTCACCTACCGAC-3′; and the forward primer, 5′-TAATTTTAGGTTAGAGGGTTATTGT-3′ and reverse primer, 5′-CACAACCAATCAACAACACA-3′ were used for the amplification of a fragment corresponding to the *CDH1* unmethylated sequence, as in Herman et al^40^. The PCR reaction was conducted using Platinum Taq High Fidelity DNA polymerase (ThermoFisher Scientific, Waltham, MA) with 40 cycles of denaturation at 94 °C for 15 seconds, annealing at 60 °C for 30 seconds and extension at 72 °C for 1 minute, following the manufacturers’ instructions.

### *CDH1* promoter methylation assessment by digital droplet PCR

The methylation status of two CpG islands in the *CDH1* promoter was assessed by ddPCR. Optimization was done with known positive controls Universal Unmethylated DNA (Millipore, Burlington, MA) and CpG Methylated DNA (ThermoFisher, Waltham, MA). Following PicoGreen quantification, 0.2–9 ng of bisulfite-treated DNA was combined with locus-specific primers (Supplementary Table S2), FAM- and HEX-labeled probes, HaeIII, and digital PCR Supermix for probes (no dUTP). All reactions were performed on a QX200 ddPCR system (Bio-Rad catalog # 1864001) and each sample was evaluated using technical duplicates. Reactions were divided into an average of ~41 K droplets per well using the QX200 droplet generator. Emulsified PCRs were run on a 96-well thermal cycler using cycling conditions defined during the optimization step (95 °C 10’; 50 cycles of 94 °C 60’ and 54 °C 2’; 98 °C 10’).

Plates were read and analyzed using the QuantaSoft software to assess the number of droplets positive for *CDH1* methylated, unmethylated, both, or neither. Samples with <300 methylated + unmethylated droplets were considered nonevaluable. Methylation Frequency (MF) = 100 * Methylated /(Methylated + Unmethylated) and those cases with an MF > 2.25 for probe 1 and/or probe 2 were considered methylated by ddPCR. Samples were considered methylated if evidence of *CDH1* gene promoter methylation was observed by either of the two methods used (i.e. MSP and/or ddCPR).

### CRISPR-Cas9 *AXIN2* knockout

Three sgRNAs against *AXIN2* were designed using CRISPick (Broad Institute, Cambridge, MA) shown in Supplementary Table S3. Presence of mutations introduced by CRISPR gene editing were assessed by Sanger sequencing with the primers indicated in Supplementary Table S4.

The sgRNAs were cloned in LentiCRISPR-v2 (Addgene #52961) and MCF7 cells were transduced with lentiviral particles produced at MSK Gene Editing & Screening Core Facility and then subjected to selection for 14 days with puromycin (2 µg/ml; ThermoFisher Scientific; Waltham, MA). NT sgRNAs were used as controls. The mutations introduced by CRISPR-Cas9 gene editing were confirmed by CRISPR-sequencing and Sanger sequencing (Supplementary Fig. S3).

### qRT-PCR

Total RNA was reverse-transcribed into cDNA using SuperScript VILO Master Mix (ThermoFisher Scientific), according to the manufacturers’ instructions. Quantitative TaqMan RT-PCR for *AXIN2* (Hs01063170_m1) and *CDH1* (Hs01023895_m1 or Hs01023894_m1) was performed using QuantStudio3 (Applied Biosystems; ThermoFisher Scientific). Expression data were normalized to *GAPDH* (Hs02786624), as previously described^41^.

### Scratch wound healing assay

Cells were seeded in 24-well plates at ~90% confluence. Twenty-four hours later a scratch was made across the center of the well with a pipette tip. Phase-contrast images were obtained immediately (0 h), and 24 h and 48 h later using an EVOS XL Core Microscope (ThermoFisher Scientific). The percentage of wound closure was defined using ImageJ, as previously described^41^. Experiments were performed in triplicates in at least 3 independent experiments.

### Cell growth in low attachment

To assess cell viability in low attachment conditions, 250,000 MCF-7 cells were seeded in 6-well low attachment plates (Corning, NY, USA). Forty-eight hours later, cell viability was assessed using trypan-blue staining. Non-viable cells were excluded and living cells were quantified using an automatic counter (Countless II, ThermoFisher, Waltham, MA, USA). Cells were reseeded in 96 well plates and cell viability was assessed 72 hours later using the CellTiter-Blue Assay (Promega), as described^41^. Absorbance was detected (560 nm excitation and 590 nm emission) using a Victor X4 Multimode Plate Reader (PerkinElmer, Hopkinton, MA, USA). Experiments were performed in triplicates in at least 3 independent experiments.

### Three-dimensional cell culture and immunofluorescence

Coverslip chambered slide wells (Ibidi # 80826) were coated with growth factor reduced (GFR) basement membrane matrigel matrix (100 μL; Corning # 356231) as previously described^42^. 3,000-5,000 MCF7 cells were resuspended in 200 µL of assay media (with 2% Matrigel) and plated on top of the Matrigel coat. Fresh assay media was replenished every 3 days and cells were cultured for 21 days. MCF7 cells grown on 3D Matrigel culture were fixed with 4% formaldehyde (ThermoFisher Scientific) and incubated with 10% normal Goat Serum (Vector Laboratories, Burlingame, CA, USA #S-1000)^41^.

E-cadherin expression was assessed using an antibody labeled with Alexa-488 (E-Cadherin (#3199) Cell signaling, MA, USA; dilution 1:200). DAPI (Abcam # ab228549) was used as nuclear counterstaining. Fluorescent images were acquired using a TCS SP5 inverted confocal microscope (Leica Microsystems, Buffalo Grove, IL, USA), as described previously^37,41,42^. No post-acquisition processing was performed, besides minor adjustments of brightness and contrast, applied equally to all images. ImageJ software was used to assess E-cadherin expression in at least five representative images per condition.

### Whole-genome sequencing

Tumor and normal DNA derived from microdissected FFPE samples of three ILCs lacking *CDH1* bi-allelic genetic or epigenetic inactivation by targeted sequencing and *CDH1* gene promoter assessment were subjected to WGS at the MSK Integrated Genomics Operations (IGO) using validated protocols, as described^43,44^. WGS was conducted with a mean sequencing coverage depth of 63x and 37x from tumor and normal samples genome-wide, respectively. Sequence reads were aligned to the reference human genome GRCh37/hg19 using the Burrows-Wheeler Aligner (BWA v0.7.15).^45^ MuTect (v1.0)^46^ was used for the detection of somatic single nucleotide variants (SNVs), whereas insertion and deletions (indels) were detected using Strelka (v2.0.15),^47^ VarScan2 (v2.3.7),^48^ Platypus (v0.8.1)^49^ and Scalpel (v0.5.3).^50^ FACETS^51^ was used for the determination of copy number alterations and loss of heterozygosity of the wild-type allele (LOH). The cancer cell fraction (CCF) of each mutation was inferred using ABSOLUTE.^52^ Mutations were considered clonal when their probability of being clonal was >50%^53^ or if the 95% confidence interval lower bound of its CCF was >90%^54,55^. Single base substitution (SBS) and indel mutational signatures (COSMIC v.3.1) were inferred using and Signal^56^ and Sigprofiler^57^ using the SigProfilerExtractor Package, respectively. Structural variants (SVs) were determined using the combination of Manta,^58^ SvABA,^59^ and GRIDSS2^60^, as previously described.^43^ SVs were combined based on the SV type, strand and constraining the genomic coordinates of breakpoint to 0.5 kbp. The signature.tools.lib R package^56^ was used for the generation of circos plots depicting SV calls, SNVs, indels and copy number alterations. HRD was assessed using HRDetect^61^.

### Reverse transcription PCR (RT-PCR) for fusion gene validation

RNA was reverse-transcribed to cDNA using SuperScript VILO Master Mix (Life Technologies; Thermo Fisher Scientific), following manufacturers’ instructions. PCR amplification of 10 ng of cDNA was conducted using a primer sets designed based in the predicted fusion gene (forward primer: ACAATACTGGGCCACATGCT; reverse primer: ccagaggacgatgaagagga) as previously described^41^. PCR fragments were running in agarose electrophoresis to confirm amplification.

### Comparison of repertoire of genetic alterations in ILC according to *CDH1* status

We compared the TMB, FGA and frequency of oncogenic/likely oncogenic somatic genetic alterations in ILCs lacking *CDH1* genetic alterations and ILCs harboring biallelic genetic inactivation of *CDH1* as identified by targeted sequencing using MSK-IMPACT. To compare the frequency of somatic genetic alterations, we used the two-tailed Fisher’s exact test. We performed multiple testing correction using the Benjamini-Hochberg procedure to control for false discovery rate. TMB and FGA were compared using the Mann-Whitney *U* test.

### Reporting summary

Further information on research design is available in the Nature Research Reporting Summary linked to this article.

### Supplementary information

REPORTING SUMMARY

Supplementary Information

## Data Availability

Somatic mutations, copy number alterations and structural variants identified by targeted sequencing are available on cBioPortal (https://www.cbioportal.org/study/summary?id=ilc_msk_2023).

## References

[1] Lokuhetty D, White V, Watanabe R, Cree I (2019). WHO Classification of Tumours Editorial Board. Breast tumors. WHO Classificat. Tumours Series, 5th ed.; Int. Agency Res. Cancer: Lyon, France.

[2] McCart Reed AE, Kalinowski L, Simpson PT, Lakhani SR (2021). Invasive lobular carcinoma of the breast: the increasing importance of this special subtype. Breast Cancer Res..

[3] Rakha EA, Ellis IO (2010). Lobular breast carcinoma and its variants. Semin. Diagn. Pathol..

[4] Weigelt B (2010). The molecular underpinning of lobular histological growth pattern: a genome-wide transcriptomic analysis of invasive lobular carcinomas and grade- and molecular subtype-matched invasive ductal carcinomas of no special type. J. Pathol..

[5] Ciriello G (2015). Comprehensive Molecular Portraits of Invasive Lobular Breast Cancer. Cell.

[6] Caldeira JR (2006). CDH1 promoter hypermethylation and E-cadherin protein expression in infiltrating breast cancer. BMC Cancer.

[7] Lombaerts M (2006). E-cadherin transcriptional downregulation by promoter methylation but not mutation is related to epithelial-to-mesenchymal transition in breast cancer cell lines. Br. J. Cancer.

[8] Pareja F (2020). The genomic landscape of metastatic histologic special types of invasive breast cancer. NPJ Breast Cancer.

[9] de Groot JS (2018). alphaE-catenin is a candidate tumor suppressor for the development of E-cadherin-expressing lobular-type breast cancer. J. Pathol..

[10] Cheng DT (2015). Memorial Sloan Kettering-Integrated Mutation Profiling of Actionable Cancer Targets (MSK-IMPACT): A Hybridization Capture-Based Next-Generation Sequencing Clinical Assay for Solid Tumor Molecular Oncology. J. Mol. Diagn..

[11] Alsaleem M (2019). The molecular mechanisms underlying reduced E-cadherin expression in invasive ductal carcinoma of the breast: high throughput analysis of large cohorts. Mod. Pathol..

[12] Mahler-Araujo B, Savage K, Parry S, Reis-Filho JS (2008). Reduction of E-cadherin expression is associated with non-lobular breast carcinomas of basal-like and triple negative phenotype. J. Clin. Pathol..

[13] Lustig B (2002). Negative feedback loop of Wnt signaling through upregulation of conductin/axin2 in colorectal and liver tumors. Mol. Cell Biol..

[14] Beard C, Purvis R, Winship IM, Macrae FA, Buchanan DD (2019). Phenotypic confirmation of oligodontia, colorectal polyposis and cancer in a family carrying an exon 7 nonsense variant in the AXIN2 gene. Fam. Cancer.

[15] Jensen JM (2022). Familial colorectal cancer and tooth agenesis caused by an AXIN2 variant: how do we detect families with rare cancer predisposition syndromes?. Fam. Cancer.

[16] Macklin-Mantia SK (2020). Case report expanding the germline AXIN2- related phenotype to include olfactory neuroblastoma and gastric adenoma. BMC Med. Genet..

[17] Boguslavsky S (2007). p120 catenin regulates lamellipodial dynamics and cell adhesion in cooperation with cortactin. Proc. Natl. Acad. Sci. USA.

[18] Short SP (2017). p120-Catenin is an obligate haploinsufficient tumor suppressor in intestinal neoplasia. J. Clin. Invest..

[19] Holliday DL, Speirs V (2011). Choosing the right cell line for breast cancer research. Breast Cancer Res..

[20] Derksen PW (2006). Somatic inactivation of E-cadherin and p53 in mice leads to metastatic lobular mammary carcinoma through induction of anoikis resistance and angiogenesis. Cancer Cell.

[21] Schackmann RC (2011). Cytosolic p120-catenin regulates growth of metastatic lobular carcinoma through Rock1-mediated anoikis resistance. J. Clin. Invest..

[22] Bucker, L. & Lehmann, U. CDH1 (E-cadherin) Gene Methylation in Human Breast Cancer: Critical Appraisal of a Long and Twisted Story. *Cancers (Basel)* **14** (2022). 10.3390/cancers14184377 10.3390/cancers14184377 PMC9497067 36139537

[23] Cortez LM (2019). APOBEC3A is a prominent cytidine deaminase in breast cancer. PLoS Genet..

[24] Petljak M (2019). Characterizing Mutational Signatures in Human Cancer Cell Lines Reveals Episodic APOBEC Mutagenesis. Cell.

[25] Swanton C, McGranahan N, Starrett GJ, Harris RS (2015). APOBEC Enzymes: Mutagenic Fuel for Cancer Evolution and Heterogeneity. Cancer Discov..

[26] Yadav S (2021). Germline Pathogenic Variants in Cancer Predisposition Genes Among Women With Invasive Lobular Carcinoma of the Breast. J. Clin. Oncol..

[27] Piersma B, Bank RA, Boersema M (2015). Signaling in Fibrosis: TGF-beta, WNT, and YAP/TAZ Converge. Front. Med. (Lausanne).

[28] Borst P, Jonkers J, Rottenberg S (2007). What makes tumors multidrug resistant?. Cell Cycle.

[29] Bossart EA (2019). SNAIL is induced by tamoxifen and leads to growth inhibition in invasive lobular breast carcinoma. Breast Cancer Res. Treat..

[30] Batlle E (2000). The transcription factor Snail is a repressor of E-cadherin gene expression in epithelial tumour cells. Nat. Cell Biol..

[31] Gall TMH, Frampton AE (2013). Gene of the month: E-cadherin (<em>CDH1</em&gt).. J. Clin. Pathol..

[32] Derksen PW (2011). Mammary-specific inactivation of E-cadherin and p53 impairs functional gland development and leads to pleomorphic invasive lobular carcinoma in mice. Dis. Model Mech..

[33] WHO Classification of Tumors Editorial Board. *Breast tumours. WHO Classification of Tumors. 5th Edition*. 5th Edition edn, (Lyon, 2019).

[34] Elston CW, Ellis IO (2002). Pathological prognostic factors in breast cancer. I. The value of histological grade in breast cancer: experience from a large study with long-term follow-up. Histopathology.

[35] Da Silva L (2008). Aberrant expression of E-cadherin in lobular carcinomas of the breast. Am. J. Surg. Pathol..

[36] Choi YJ, Pinto MM, Hao L, Riba AK (2008). Interobserver variability and aberrant E-cadherin immunostaining of lobular neoplasia and infiltrating lobular carcinoma. Mod. Pathol..

[37] Pareja F (2018). Loss-of-function mutations in ATP6AP1 and ATP6AP2 in granular cell tumors. Nat. Commun..

[38] da Silva EM (2021). TERT promoter hotspot mutations and gene amplification in metaplastic breast cancer. NPJ Breast Cancer.

[39] Chiang S (2016). IDH2 Mutations Define a Unique Subtype of Breast Cancer with Altered Nuclear Polarity. Cancer Res..

[40] Herman JG, Graff JR, Myohanen S, Nelkin BD, Baylin SB (1996). Methylation-specific PCR: a novel PCR assay for methylation status of CpG islands. Proc. Natl. Acad. Sci. USA.

[41] Kim SH (2022). Recurrent WWTR1 S89W mutations and Hippo pathway deregulation in clear cell carcinomas of the cervix. J Pathol..

[42] Geyer FC (2018). Recurrent hotspot mutations in HRAS Q61 and PI3K-AKT pathway genes as drivers of breast adenomyoepitheliomas. Nat. Commun..

[43] Selenica P (2022). APOBEC mutagenesis, kataegis, chromothripsis in EGFR-mutant osimertinib-resistant lung adenocarcinomas. Ann. Oncol..

[44] Riaz N (2021). Precision Radiotherapy: Reduction in Radiation for Oropharyngeal Cancer in the 30 ROC Trial. J. Natl. Cancer Inst..

[45] Li H, Durbin R (2010). Fast and accurate long-read alignment with Burrows-Wheeler transform. Bioinformatics.

[46] Cibulskis K (2013). Sensitive detection of somatic point mutations in impure and heterogeneous cancer samples. Nat. Biotechnol..

[47] Saunders CT (2012). Strelka: accurate somatic small-variant calling from sequenced tumor-normal sample pairs. Bioinformatics.

[48] Koboldt DC (2012). VarScan 2: somatic mutation and copy number alteration discovery in cancer by exome sequencing. Genome Res.

[49] Rimmer A (2014). Integrating mapping-, assembly- and haplotype-based approaches for calling variants in clinical sequencing applications. Nat. Genet..

[50] Narzisi G (2014). Accurate de novo and transmitted indel detection in exome-capture data using microassembly. Nat. Methods.

[51] Shen R, Seshan VE (2016). FACETS: allele-specific copy number and clonal heterogeneity analysis tool for high-throughput DNA sequencing. Nucleic Acids Res..

[52] Carter SL (2012). Absolute quantification of somatic DNA alterations in human cancer. Nat. Biotechnol..

[53] Landau DA (2013). Evolution and impact of subclonal mutations in chronic lymphocytic leukemia. Cell.

[54] Martelotto LG (2017). Whole-genome single-cell copy number profiling from formalin-fixed paraffin-embedded samples. Nat. Med..

[55] Ng CKY (2017). The Landscape of Somatic Genetic Alterations in Metaplastic Breast Carcinomas. Clin. Cancer Res..

[56] Degasperi A (2020). A practical framework and online tool for mutational signature analyses show inter-tissue variation and driver dependencies. Nat. Cancer.

[57] Islam SMA (2022). Uncovering novel mutational signatures by de novo extraction with SigProfilerExtractor. Cell Genom..

[58] Chen X (2016). Manta: rapid detection of structural variants and indels for germline and cancer sequencing applications. Bioinformatics.

[59] Wala JA (2018). SvABA: genome-wide detection of structural variants and indels by local assembly. Genome Res..

[60] Cameron DL (2021). GRIDSS2: comprehensive characterisation of somatic structural variation using single breakend variants and structural variant phasing. Genome Biol..

[61] Davies H (2017). HRDetect is a predictor of BRCA1 and BRCA2 deficiency based on mutational signatures. Nat. Med..

