# Supplementary material for: Genomic and epigenomic basis of breast invasive lobular carcinomas lacking CDH1 genetic alterations
Source: NPJ Precis Oncol. 2024 Feb 12;8:33. doi: 10.1038/s41698-024-00508-x (PMC10861500; doi:10.1038/s41698-024-00508-x)
Supplement: Supplementary file 2 — Supplementary Information [file 41698_2024_508_MOESM2_ESM.pdf]

SUPPLEMENTARY INFORMATION

Genomic and Epigenomic Basis of Breast Invasive Lobular Carcinomas Lacking *CDH1* Genetic Alterations

Dopeso et al.

Supplementary Figures S1-S7
Supplementary Table S1-S4

Supplementary Figure S1

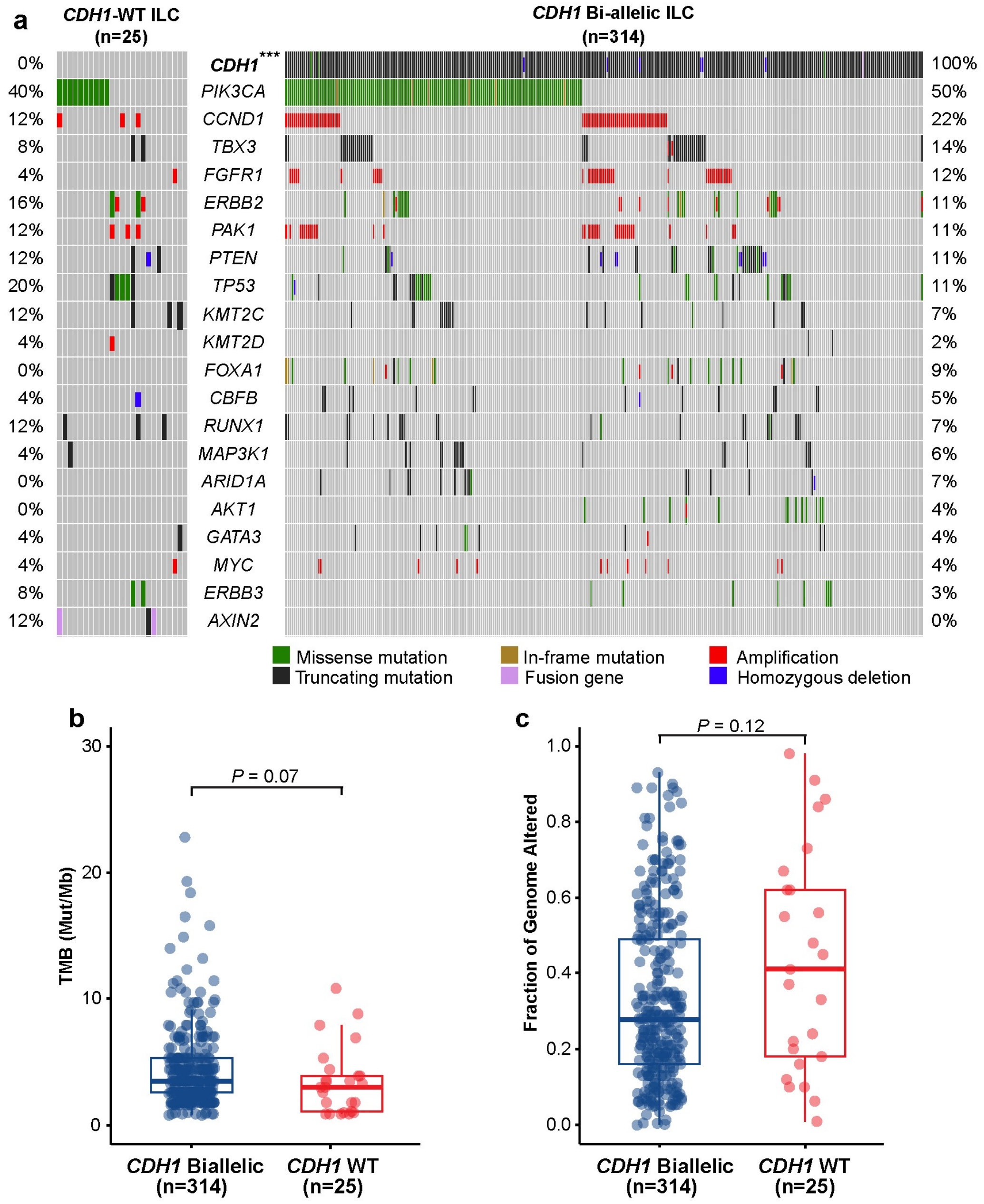

Supplementary Figure S1. Comparison of genetic alterations between invasive lobular carcinomas (ILCs) according to *CDH1* status (**a**) Heatmap depicting the frequency of oncogenic/likely oncogenic genetic alterations in ILCs lacking *CDH1* genetic alterations (n=25) and ILCs harboring biallelic *CDH1* genetic inactivation (n=314). ****P*<0.001; Fisher's exact test with Benjamini-Hochberg multiple-testing correction. (**b-c**) Boxplots depicting the (**b**) tumor mutation burden (TMB) and (**c**) fraction of genome altered (FGA) in ILCs with biallelic *CDH1* genetic inactivation (n=314) and ILCs lacking *CDH1* genetic alterations (n=25). Mann Whitney *U*-test.

Supplementary Figure S2

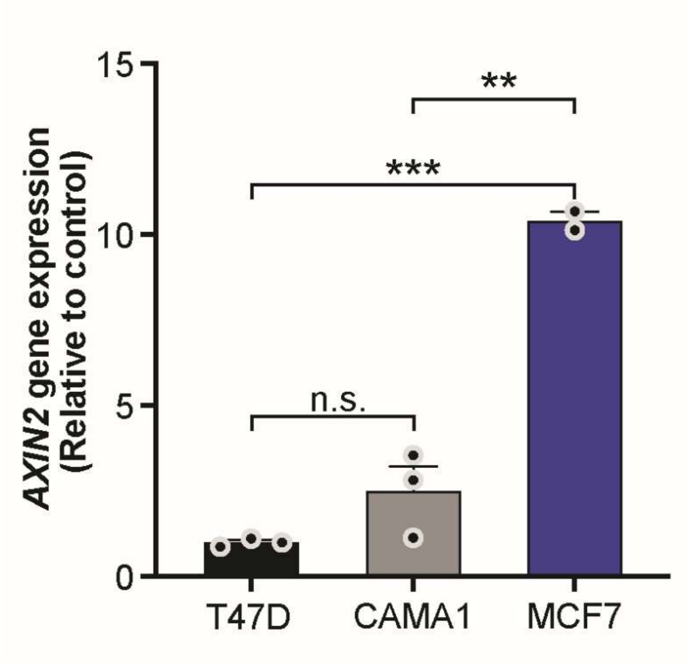

Supplementary Figure S2. *AXIN2* expression in breast cancer cell models. *AXIN2* expression by RT-PCR in the estrogen receptor (ER)-positive/HER2-negative breast cancer cell models T47D, CAMA1 and MCF7. n.s., not significant. ***P*<0.01; ****P*<0.001; two-tailed t-test.

Supplementary Figure S3

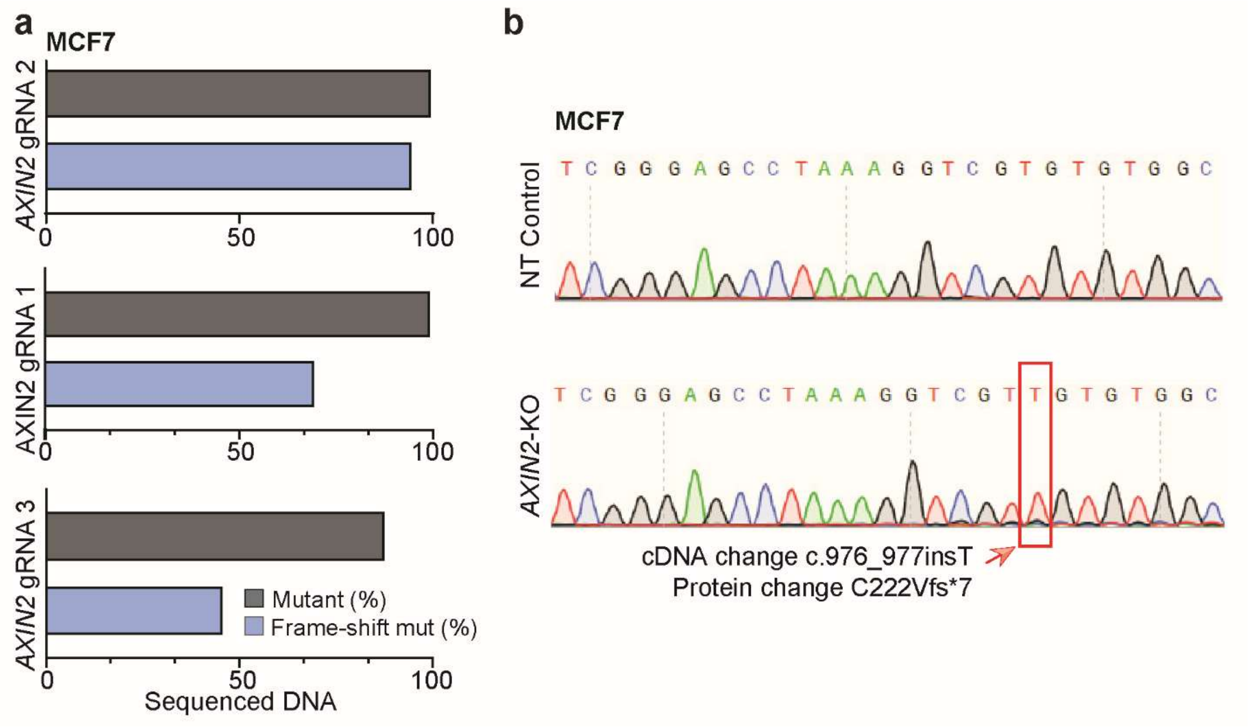

Supplementary Figure S3. Assessment of *AXIN2* CRISPR gene editing by CRISPR screening and Sanger sequencing. (a) Percentage of mutated or frameshift-mutated DNA in MCF-7 *AXIN2*-Knockout (KO) cells assessed by CRISPR-screening. (**b**) Representative Sanger sequencing electropherograms of *AXIN2*-KO (gRNA2) and non-target (NT) control MCF-7 cells.

Supplementary Figure S4

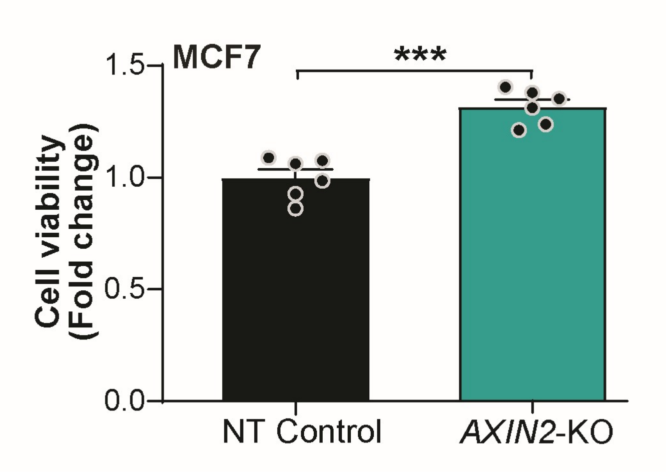

Supplementary Figure S4. Anoikis resistance in MCF7 cells with *AXIN2* loss of function. Cell viability of *AXIN2*-KO and non-target (NT) control MCF7 cells upon seeding in adherent conditions following 72 hours of cell culture in low attachment conditions. ****P*<0.001; two-tailed t-test.

Supplementary Figure S5

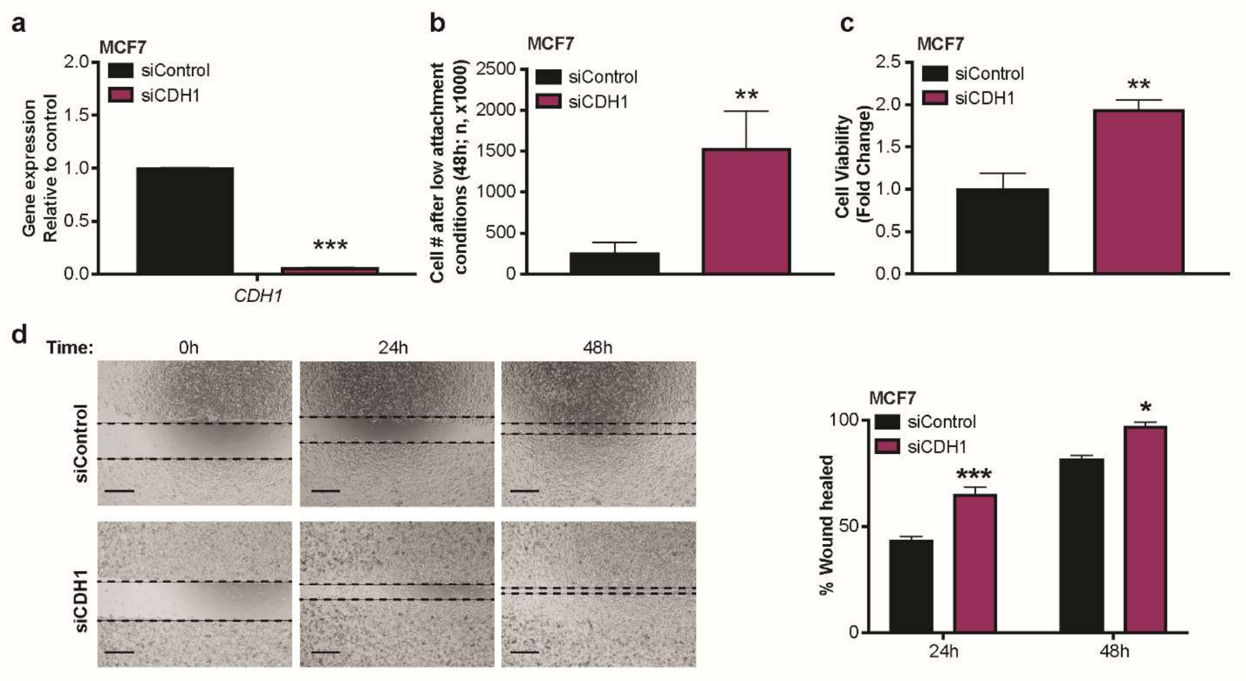

Supplementary Figure S5. *CDH1* inactivation results in the acquisition of lobular-like features in MCF7 breast cancer cell models (**a**) Quantitative assessment of *CDH1* expression in MCF7 cells in which *CDH1* was silenced with siRNAs (siCDH1) compared to siRNA Control (siControl) by RT-PCR. (**b**) Number of siCDH1 MCF7 cells compared to siControl following culture in non-adherent (low attachment) conditions for 48 hours. (**c**) Cell viability assay of siCDH1 and siControl MCF7 cells after culture in non-adherent conditions. (**d**) Wound healing assay of siCDH1 MCF7 cells compared to siRNA control. Wound area was assessed at 0, 24 and 48 hours. Representative micrographs (*left*) and wound closure quantification (*right*) are depicted. Scale bar, 500 µm. Data are representative of at least three independent experiments. *$P < 0.05$, **$P < 0.01$, ***$P < 0.001$; two-tailed *t*-test.

Supplementary Figure S6

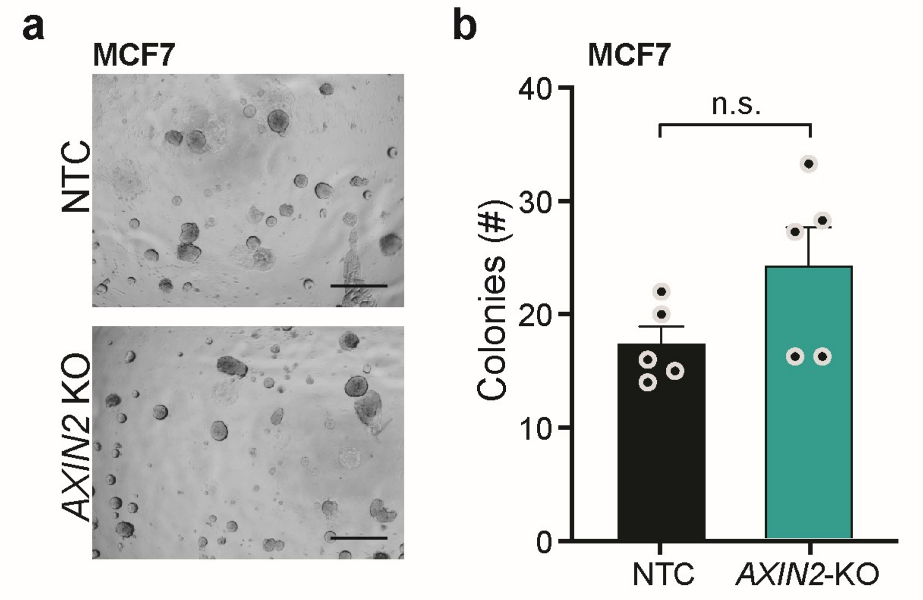

Supplementary Figure S6. Colony formation of *AXIN2*-knock out and non-target control MCF7 3D cell models. (**a**) Representative micrographs of *AXIN2*-knock out (KO) and non-target (NT) control MCF7 cells grown on 3D cell culture. (**b**) Quantification of the number of colonies per well in *AXIN2*-KO and NT control MCF-7 cells grown on 3D cell culture. n.s., non-significant; two-tailed t-test.

Supplementary Figure S7

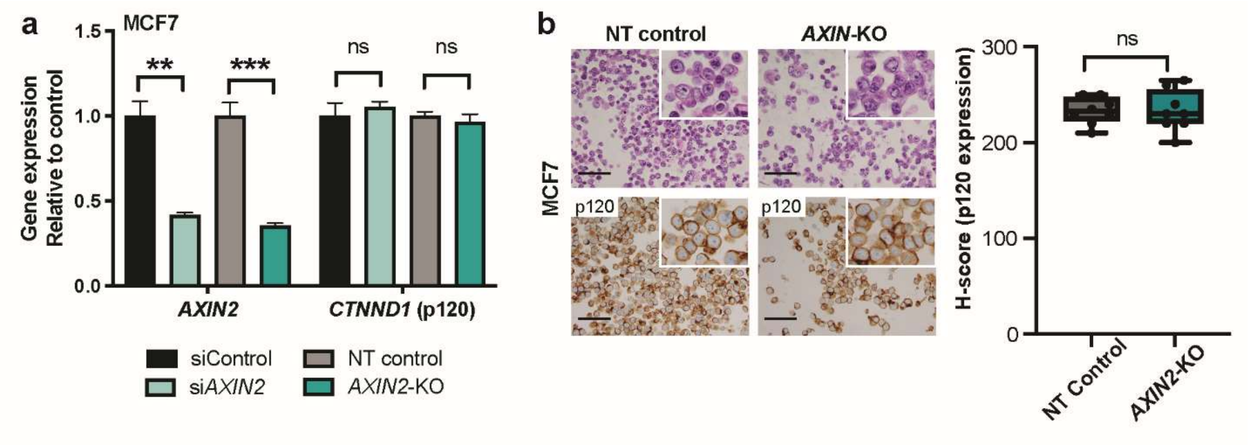

Supplementary Figure S7. *CTNND1* (p120) expression upon *AXIN2* depletion.
(a) *AXIN2* and *CTNND1* (p120) mRNA expression by RT-PCR in *AXIN2*-knock out (KO) and non-target (NT) control MCF7 cells, and upon silencing of *AXIN2* with siRNAs and in siRNA control cells. Data are representative of at least three independent experiments. (**b**) Representative micrographs depicting p120 (*CTNND1*) expression in formalin fixed paraffin embedded (FFPE) *AXIN2*-KO and MCF7 cell pellets by immunohistochemistry (*left*). Boxplots depicting p120 protein expression (H-score) in NT control and AXIN2-KO MCF7 cells *(right)*. n.s., not significant; $^{**}P < 0.01$: $^{***}P < 0.001$; two-tailed unpaired *t*-test.

Supplementary Table S1. E-cadherin protein expression by immunohistochemistry in primary invasive lobular carcinomas according to *CDH1* genetic status.

**E-cadherin expression**	***CDH1* genetic status as per targeted sequencing (MSK-IMPACT)**			
	***CDH1* biallelic genetic inactivation (n=211)**	***CDH1* monoallelic genetic inactivation (n=16)**	**No *CDH1* genetic alterations (n=18)**	**Total**
Negative	195 (92.4%)	15 (93.8%)	14 (77.8%)	224 (91.4%)
Decreased	6 (2.8%)	0 (0.0%)	2 (11.1%)	8 (3.3%)
Aberrant	10 (4.7%)	1 (6.3%)	1 (5.6%)	12 (4.9%)
Retained	0 (0.0%)	0 (0.0%)	1 (5.6%)	1 (0.4%)
**Total**	211 (100.0%)	16 (100.0%)	18 (100.0%)	245 (100.0%)

Supplementary Table S2. Locus specific primers for *CDH1* promoter methylation assessment by digital droplet PCR

Assay name	Forward sequence	Reverse sequence	Probe sequence	Fluorophore
CDH1_1_NonMethyl	AGTAATTTTAGGTTAGAGGGTT	AAATTCACCTACCAACCAC	TTGTGTTTATGTGAGGTTGG	HEX
CDH1_2_Methyl	TGCGGAAGTTAGTTTAGATT	AACCCATAACTAACCGAAAA	CGTTTTAGTTCGGTTCGATT	FAM
CDH1_2_NonMethyl	TTTGTGGAAGTTAGTTTAGATTT	CTAACCAAAAACACCAAACA	TGATTGTATTTGGTGTTTGTTT	HEX
CDH1_1_methyl	AGTAATTTTAGGTTAGAGGGTT	GACCACAACCAATCAACA	CGTTAGTTTCGTTTTGGGG	FAM

Supplementary Table S3. Single guide RNAs utilized for *AXIN2* knockout

Gene	Target Transcript	Orientation	sgRNA Sequence	Exon
AXIN2	NM_004655.4	Antisense	GGGAGTGGTACTGCGAATGG	6
AXIN2	NM_004655.4	Sense	GGGAGCCTAAAGGTCGTGTG	2
AXIN2	NM_004655.4	Sense	GCTCACACTCAATTCGCGGG	6

Supplementary Table S4. Primers used for assessment of *AXIN2* CRISPR gene editing by Sanger Sequencing

**Forward primer**	**Sequence**	**Reverse primer**	**Sequence**
AXIN2_T1_F	CACCGGGAGTGGTACTGCGAATGG	AXIN2_T1_R	AAACCCATTCGCAGTACCACTCCC
AXIN2_T2_F	CACCGGGAGCCTAAAGGTCGTGTG	AXIN2_T2_R	AAACCACACGACCTTTAGGCTCCC
AXIN2_T3_F	CACCGCTCACACTCAATTCGCGGG	AXIN2_T3_R	AAACCCCGCGAATTGAGTGTGAGC
